# Correlates of mental health of people living with HIV co-infected with SARS-CoV-2: findings from the COVIDHIV study in France

**DOI:** 10.3389/fpsyt.2024.1437362

**Published:** 2025-01-03

**Authors:** Issifou Yaya, Yvenie Amboise, Guillaume Roucoux, Lisa Yombo-Kokule, Fabienne Marcellin, Claudine Duvivier, Karine Lacombe, James W. Griffith, Marie Préau, Antoine Cheret, Martin Duracinsky

**Affiliations:** ^1^ Patient-Reported Outcomes Research (PROQOL), Health Economics Clinical Trial Unit (URC-ECO), Hotel-Dieu Hospital, AP- HP, Paris, France; ^2^ ECEVE, UMR-S 1123, Paris Cité University, Inserm, Paris, France; ^3^ Aix Marseille Univ, Inserm, IRD, SESSTIM, Sciences Economiques et Sociales de la Santé et Traitement de l’Information Médicale, ISSPAM, Marseille, France; ^4^ Paris Cité University, Necker Hospital, AP-HP, Infectious Diseases Department, Necker-Pasteur Infectiology Center, Paris, France; ^5^ Inserm U1016, CNRS UMR 8104, Université Paris Descartes, Institut Cochin, Paris, France; ^6^ Hôpital Saint-Antoine, Assistance Publique Des Hôpitaux de Paris, Service Des Maladies Infectieuses Et Tropicales, Cedex 12, Paris, France; ^7^ INSERM, Pierre Louis Institute of Epidemiology and Public Health (IPLESP), Sorbonne University, Paris, France; ^8^ Department of Obstetrics and Gynecology, Biological Sciences Division, The University of Chicago, Chicago, IL, United States; ^9^ Department of Psychiatry and Behavioral Neuroscience, Biological Sciences Division, The University of Chicago, Chicago, IL, United States; ^10^ Inserm Unit 1296 « Radiations: Defense, Health, Environment »; Lyon 2 Lumière University, Lyon, France; ^11^ Plateforme de Diagnostic et de Thérapeutique Pluridisciplinaire, CHU, Guadeloupe, France; ^12^ INSERM, U1016, CNRS, UMR8104, Institut Cochin, Paris, France; ^13^ Internal Medicine Unit, Le Kremlin Bicêtre Hospital, Bicêtre, France

**Keywords:** COVID-19, HIV, anxiety, depression, post-traumatic stress disorder, mental health

## Abstract

**Background:**

The COVID-19 pandemic has severely affected vulnerable populations, especially individuals living with HIV/AIDS (PLWHA). The convergence of HIV/AIDS and COVID-19 presents unique challenges, exacerbating existing health concerns and magnifying the strain on individuals already grappling with compromised immune systems. This study aimed to investigate the mental well-being repercussions faced by PLWHA co-infected with SARS-CoV-2 in France.

**Methods:**

COVIDHIV is a French multicenter cohort of PLWHA co-infected with SARS-CoV-2, which collected sociodemographic, clinical, and mental health data. Anxiety and depression symptoms and post-traumatic stress disorder (PTSD) were assessed by the Hospital Anxiety and Depression Scale (HADS) and the PTSD Checklist (PCL-S), respectively. Multivariable logistic regression was performed to identify factors associated with mental health outcomes at inclusion in the cohort.

**Results:**

Of the 397 participants included, 64.7% were male. The mean age was 51.6 (± 11.8) years. The prevalence of mental health outcomes was 33.5% ([95%CI: 28.5-39.0%]) for anxiety, 21.0% ([16.8-25.9%]) for depression, and 12.2% ([8.9-16.5%]) for PTSD. In multivariable regression adjusted for sex, COVID-19 wave and duration between COVID-19 confirmation and enrolment, age (adjusted odds-ratio (aOR): 0.97 [0.95-0.99]), being professionally active (0.43 [0.25-0.75]), and the number of self-reported symptoms (1.17 [1.11-1.24]) were associated with anxiety. Being professionally active (0.34 [0.18-0.65]), living in a couple (0.52 [0.20-0.98]), number of self-reported symptoms (1.15 [1.08-1.22]), and hospitalization for COVID-19 (3.35 [1.34-8.33]) were associated with depression. The number of self-reported symptoms (1.27 [1.16-1.41]), psychiatric disorders (4.04 [1.48-11.11]), and perceived vulnerability to COVID-19 (4.53 [1.69-14.60]) were associated with PTSD.

**Conclusion:**

The mental health is a challenging issue among the participants and needs to be closely monitored among people already affected by a chronic disease such as HIV. The findings underscore the urgent need for targeted support and interventions tailored to address the mental health needs of PLWHA facing the dual burden of HIV/AIDS and COVID-19.

## Background

The COVID-19 pandemic has had profound effects on public health, economies, and daily life worldwide. COVID-19 resulted in about seven million deaths worldwide as of March 2023 (1, 2). COVID-19 affected disproportionally regions but also individuals. In fact, it could cause severe respiratory distress in vulnerable populations, including older people, those with underlying medical comorbidities, and those living with HIV infection. These populations were at higher risk of poor COVID-19 outcomes (3–5). Coinfection with COVID-19 in people living with HIV/AIDS (PLWHA) may have particular clinical and epidemiological implications, due to their abnormal humoral and cellular immunity (6, 7). Besides, prior the COVID-19 pandemic, PLWHA already experienced significant stigma, discrimination, and social isolation due to their condition, making them particularly vulnerable to the mental health challenges posed by the pandemic (8). Both COVID-19 and HIV significantly affect mental health, with the intersection of PTSD, HIV, and COVID-19 being particularly complex, involving psychological, immunological, and social factors. PLWHA are at higher risk of PTSD due to chronic stress from managing their condition, compounded by stigma and health challenges. This stress, worsened by a COVID-19 infection, can intensify PTSD symptoms and hinder recovery, as HIV weakens immune function (9, 10). This stress can worsen PTSD symptoms and complicate recovery due to HIV’s effects on immune function, while PTSD itself may further complicate HIV and COVID-19 management, leading to poor treatment adherence and comorbidities like depression (11, 12).

The COVID-19 pandemic also introduced widespread stressors, such as social isolation, financial difficulties, and disrupted daily routines, contributing to increased anxiety, depression, and overall mental health challenges (9, 13). In many countries, restrictions on healthcare services during the pandemic, including mental health support, exacerbated the risk of mental health deterioration (14, 15) (15). Additionally, COVID-19 disrupted essential HIV services, affecting prevention, testing, and treatment efforts globally, even in high-income settings (16, 17).

Several recent cross-sectional studies found that during the COVID-19 pandemic, PLWHA experienced increased mental health symptoms (18, 19). A recent scoping review on mental health outcomes among PLWHA during the COVID-19 pandemic, regardless of SARS-CoV-2 infection status, found that mental health problems including depressive symptoms, anxiety, and Post‐Traumatic Stress Disorder (PTSD) were commonly reported in that population (11). Another study evaluating mental health and social support factors among PLWHA in Argentina and the USA during the COVID-19 pandemic revealed higher levels of depression among them (20). In France, Parisi et al. found that 27.8% of the PLWHA had moderate or severe depression symptoms at baseline, regardless of their COVID-19 status (18). It was also highlighted that these mental health conditions were associated with substance use, lack of antiretroviral adherence, social support, financial stress, and economic vulnerability during the COVID-19 pandemic (11, 20).

The COVID-19 pandemic highlighted the importance of integrated care for PLWHA and addressing their mental health needs. However, to the best of our knowledge, no study has investigated mental health problems, including depression, anxiety, and stress, among PLWHA co-infected with SARS-CoV-2 in France. This study examined correlates of mental health (anxiety, depression, and PTSD) among PLWHA co-infected with SARS-CoV-2 in France. Understanding these correlates is crucial for developing targeted interventions, support systems, and policies to address the mental health needs of this vulnerable population effectively. Furthermore, by filling this gap in the literature, the study may contribute to the broader understanding of the intersectionality of infectious diseases and mental health, ultimately aiding in the improvement of holistic healthcare practices and services for individuals living with complex health conditions.

## Material and methods

### Design and study population

This study was a cross-sectional from the COVIDHIV cohort in France, designed to characterize the clinical and biological characteristics of COVID-19 disease in PLWHA. One of the secondary objectives of this cohort study was to document patient-reported outcomes (including mental health) in PLWHA co-infected or not with SARS-CoV-2. From March 18^th^, 2020 to June 28^th^, 2022, adult PLWHA, including PLWHA confirmed to be infected with SARS-CoV-2 since January 1^st^, 2020, were recruited in 42 hospitals across the country, regardless of whether they met the hospitalization criteria, and the participants were followed up for one year. Infection with SARS-CoV-2 was confirmed by polymerase chain reaction (PCR), serology, imaging (CT scan/radiography), or an antigenic test. PLWHA tested positive for SARS-CoV-2 were prospectively enrolled within seven days of testing positive, whereas those known to be co-infected with SARS-CoV-2 since January 1^st^, 2020 were retrospectively enrolled.

PLWHA who did not speak French, were under the age of 18, pregnant or breastfeeding, diagnosed with a pathogen other than SARS-CoV-2, and did not have medical assurance were not included in the cohort study.

### Data collection

Clinical, biological and therapeutic data were collected from patient records using an electronic case report form (eCRF). Socio-behavioral data and patient-reported outcomes, including mental health outcomes, were collected using self-administered questionnaires. Self-reported symptoms were assessed using the modified Justice Symptom Index (JSI) questionnaire (21). The questionnaire also included specific modules developed to assess knowledge and beliefs, adherence to antiretroviral treatment, and behaviors in the context of COVID-19 transmission. These modules were tested on a subgroup of patients prior to implementation.

### Study population and outcomes

The study population included all cohort participants. The mental health study outcomes were anxiety, depression, and PTSD.

Anxiety and depression were assessed using the generic Hospital Anxiety and Depression Scale (HADS) (22). It is a self-assessment scale for anxiety and depressive symptoms, composed of 14 items consisting of two subscales to assess anxiety and depression. This questionnaire has seven items, each for measurement of anxiety and depression, with each item graded on a four-point Likert scale (from 0 to 3). The total score ranges from 0 to 21 and is interpreted as normal (0–7), borderline abnormal (8–10), and abnormal (11–21). In this analysis, a score of more than 7 was considered indicative of the presence of anxiety and depression (23). The French version of this scale has been the subject of validation work (24, 25).

The PTSD was measured using a 20-item self-report scale, the Post-Traumatic Stress Disorder Checklist, based on the DSM-5 (PCL-5) (26), created by Weathers et al. in 2013 (27) and translated into French by N. Desbiendras (28). These 20 items are divided into 4 symptom subgroups: (1) intrusion symptoms (items 1 to 5), (2) Avoidance symptoms (items 6 and 7), (3) Symptoms of altered cognition and mood (items 8 to 14), (4) Symptoms of profound changes in the state of arousal and associated reactivity (items 15 to 20). Participants symptoms were assessed using a 5-point Likert scale (0 = Not at all, 1 = A little bit, 2 = Moderately, 3 = Quite a bit, 4 = Extremely), with a total score ranging from 0 to 80. A total score ≥33 points was considered indicative of PTSD, with higher scores indicating more severe PTSD symptoms.

### Explanatory variables

In view of the associations found in the literature regarding factors related to mental health outcomes, the following potential explanatory variables were used in the analyses:

Sociodemographic: age (categorized in <40, 40-60, >60), sex (male/female), education (categorized as high school/plus and primary/no education); country of birth (born in metropolitan France); professionally active (those who reported being employed or maintaining professional activity).

HIV-related variables: HIV transmission mode (containing 5 variables: heterosexual (no/yes), homosexual & bisexual, transfusion, injection drug use, Other); marital status (living in a couple no/yes); CDC HIV classification (containing 3 categories: A=asymptomatic or acute, B= moderate symptom, C= severe symptom), CD4 count at inclusion (classified into two categories <500 cells/mm3, ≥ 500 cells/mm3); plasma viral load detectability (> 50 copies/mL), time since HIV diagnosis;

Clinical and biological characteristics: number of self-reported symptoms (continuous variable); hospitalization; presence of overweight or obesity (body mass index (BMI) ≥ 25), time between confirmation of COVID-19 diagnosis and enrolment (day); as well as co-morbidities: psychiatric disorders, respiratory diseases, cardiovascular diseases, hypertension, diabetes.

Behavioral and COVID-19-related variables: knowledge about COVID-19 (assessed using the following item: *“Do you think you have sufficient knowledge about COVID-19?”)*, psychological support (“*Have you received any psychological support in the last two weeks?*”, either from friends, family members, etc), self-perceived vulnerability (*“Do you feel more vulnerable to COVID-19 because of your HIV status?”)*, smoking, and alcohol use was calculated from the AUDIT-C score (unhealthy alcohol use was defined as alcohol consumption greater or equal to 3 drinks for women and greater or equal to 4 for men.

Moreover, our analyses were adjusted for the COVID-19 wave period in France: from March 2020 to September 2020 for the 1^st^ wave, from September 2020 to March 2021 for the 2^nd^ one, from March 2021 to July 2021 for the 3^rd^ one, from July 2021 to October 2021 for the 4^th^ one, and from October 2021 to July 2022 for the 5th wave. Besides, the participants included in each wave were different from each other.

### Statistical analyses

A descriptive analysis of mental health outcomes was performed, with continuous variables expressed as median (interquartile range (IQR)), and categorical variables as absolute frequency and percentage.

The prevalence of symptoms of anxiety, depression, and PTSD was estimated using the aforementioned cut-off scores and reported as percentages and 95% confidence intervals (CI). Chi-square tests were used to compare the prevalence of mental health outcomes between different participant groups. To explore factors potentially associated with anxiety, depression, and PTSD, univariable logistic regression and multivariable logistic regression models were performed, and unadjusted/adjusted odds-ratios (ORs/aORs) and associated 95% CIs were presented. Then, all of the variables with a p-value <0.25 in the univariable analyses were included in the multivariable model. In the multivariable model, a manual backward selection method combined with the Wald test was applied to identify variables that contributed to achieving a final parsimonious model (29, 30). The level of significance was 5%. All the statistical analyses were performed using R Studio/4.1.3 software.

### Ethical consideration

Ethical consent was obtained from the French Ethics Committee “Comité de Protection des Personnes” (CPP) Ile-de-France 8 (N°200406 of May 28^th^, 2021). The cohort study was registered at ClinicalTrials.gov (NCT04361604). Informed and written consent was collected from each participant. After data collection, a unique identifier was created for each participant based on the center number to ensure anonymity and confidentiality.

## Results

From March 2020 to June 2022, 408 PLWHA were recruited in the COVIDHIV cohort. Among them, 371 PLWHA co-infected with SARS-CoV-2 were included in the current study. Their mean age (± SD) was 51.6 ± 11.8 years, and 64.7% were men (**Table 1**). Fifty-three percent were living in couples, and 85.7% had a high school level [education] or higher. Less than half of the participants (44.1%) were born in metropolitan France.

**Table 1:** Sociodemographic, clinical, biological and behavioral characteristics of PLWHA co-infected with SARS-CoV-2 at inclusion in the French COVIDHIV cohort study.

Characteristics and comorbidity (N=371)	Overall		Anxiety			Depression			Post-Traumatic Stress disorder		
	N	n (%) or mean (±SD)	N (%) or mean (±SD)	Yes, n (%) or mean (±SD)	p-value	N (%) or mean (±SD)	Yes, n (%) or mean (±SD)	p-value	N (%) or mean (±SD)	Yes, n (%) or mean (±SD)	p-value
Sociodemographic characteristics											
**Age (years)**	371	51.6 (11.8)	310 *51.4 (±11.6)*	104 *49.8 (±12.4)*	0.099^a^	309 *49.8 (±12.4)*	65 *52.2 (±12.5)*	0.562^a^	294 *49.8 (±12.4)*	36 *50.9 (±13.4)*	0.689^a^
**Sex** *Female*	371	131 (35.3)	310 *107 (34.5)*	104 *43 (40.2)*	0.095^b^	309 *107 (34.6)*	65 *24 (22.4)*	0.771^b^	294 *101 (34.4)*	36 *18 (17.8)*	0.054^b^
**High school level or higher** *Yes*	328	279 (85.1)	273 *234 (85.7)*	90 *73 (31.2)*	0.143^c^	272 *233 (85.7)*	56 *41 (17.6)*	**0.006** ^c^	260 *223 (85.8)*	34 *28 (12.6)*	0.597^c^
**Professionally active** *Yes*	363	227 (62.5)	303 *188 (62.0)*	100 *51 (27.1)*	**0.008^b^**	302 *188 (62.3)*	62 *26 (13.8)*	**<0.001** ^b^	288 *176 (61.1)*	36 *18 (10.2)*	0.201^b^
**Born in metropolitan France** *Yes*	370	163 (44.1)	310 *145 (46.8)*	104 46 (31.7)	0.548^c^	309 *145 (46.9)*	65 *26 (17.9)*	0.263^c^	294 *136 (46.3)*	36 *15 (11.0)*	0.596^c^
**Living in a couple** *Yes*	360	190 (52.8)	302 *161 (53.3)*	101 *51 (31.7)*	0.541^c^	301 *161 (53.5)*	63 *25 (15.5)*	**0.016** ^c^	286 *153 (53.5)*	35 *13 (8.5)*	**0.047** ^c^
HIV-related characteristics											
**Mode of HIV transmission** *Heterosexual* *Homosexual & bisexual* *Blood transfusion* *Injection drug use* *Other*	362	185 (51.1) 142 (39.2) 5 (1.4) 19 (5.2) 11 (3.0)	305 *149 (48.9)* *124 (40.7)* *5 (1.6)* *18 (5.9)* *9 (3.0)*	100 *48 (32.2)* *39 (31.5)* *2 (40.0)* *9 (50.0)* *2 (22.2)*	0.539^b^	305 *149 (48.9)* *124 (40.7)* *5 (1.6)* *18 (5.9)* *9 (3.0)*	64 *30 (20.1)* *23 (18.5)* *2 (40.0)* *8 (44.4)* *1 (11.1)*	0.088^b^	289 *141 (48.8)* *118 (40.8)* *5 (1.7)* *16 (5.5)* *9 (3.1)*	36 *19 (13.5)* *11 ( 9.3)* *2 (40.0)* *3 (18.8)* *1 (11.1)*	0.261^b^
**CD4 count ≥500 cells/mm^3^** *Yes*	365	236 (64.7)	307 *203 (66.1)*	103 *68 (33.5)*	>0.999^b^	306 203 (66.3)	64 (20.9) 38 (18.7)	0.239^b^	291 *193 (66.3)*	36 *25 (13.0)*	0.814^b^
**Detectable HIV viral load (>50 copies/mL),** *Yes*	364	30 (8.2)	306 *24 (7.8)*	102 *7 (29.2)*	0.822^c^	306 24 (7.8)	64 (20.9) 4 (16.7)	0.795^c^	290 *23 (7.9)*	36 *0 (0.0)*	0.091^c^
CDC HIV clinical stage* *A* *B* *C*	365	200 (54.8%) 62 (17.0%) 103 (28.2%)	307 *175 (57.0%)* *53 (17.3%)* *79 (25.7%)*	103 *54 (30.9%)* *24 (45.3%)* *25 (31.6%)*	0.144^c^	306 *175 (57.2%)* *53 (17.3%)* *78 (25.5%)*	63 *33 (18.9%)* *17 (32.1%)* *13 (16.7%)*	0.083^c^	291 *165 (56.7)* *49 (16.8)* *77 (26.5)*	36 *18 (10.9)* *8 (16.3)* *10 (13.0)*	0.517^c^
COVID-19-related characteristics and comorbidities											
Duration of COVID19 over 7days** *Yes*	369	280 (75.9)	308 *241 (78.2)*	102 *79 (32.8)*	0.927^b^	307 *241 (78.5)*	65 *52 (21.6)*	0.872^b^	292 *228 (78.1)*	36 *32 (14.0)*	0.145^b^
**Wave of COVID-19** *Wave_1* *Wave_2* *Wave_3* *Wave_4* *Wave_5*	371	149 (40.2) 107 (28.8) 40 (10.8) 10 (2.7) 65 (17.5)	310 *127 (41.0)* *88 (28.4)* *36 (11.6)* *8 (2.6)* *51 (16.5)*	104 *39 (30.7)* *30 (34.1)* *13 (36.1)* *3 (37.5)* *19 (37.3)*	0.918^b^	309 *126 (40.8)* *88 (28.5)* *36 (11.7)* *8 (2.6)* *51 (16.5)*	65 *27 (21.4)* *20 (22.7)* *7 (19.4)* *2 (25.0)* *9 (17.6)*	0.958^b^	294 *115 (39.1)* *89 (30.3)* *35 (11.9)* *8 ( 2.7)* *47 (16.0)*	36 *19 (16.5)* *8 (9.0)* *1 (2.9)* *1 (12.5)* *7 (14.9)*	0.198^b^
**New COVID-19 reinfection** *Yes*	218	16 (7.3)	193 *13 (6.7)*	68 *5 (38.5)*	0.773^c^	192 *13 (6.8)*	39 *4 (30.8)*	0.304^c^	193 *13 (6.7)*	26 *1 (7.7)*	>0.999^c^
**Number of COVID-19-related symptoms,**	319	*7 (±5)*	307 *8 (±5)*	103 *10 (±5)*	**<0.001** ^a^	306 *8 (±5)*	64 *11 (±5)*	**<0.001** ^a^	291 *8 (±5)*	36 *13 (±5)*	**<0.001** ^a^
**Hospitalization for COVID-19** *Yes*	355	49 (13.8)	296 *30 (10.1)*	99 *13 (43.3)*	0.228^c^	295 *30 (10.2)*	62 *13 (43.3)*	**0.004** ^c^	280 *27 (9.6)*	35 *2 (7.4)*	0.549^c^
**Received instructions at hospital discharge,** *Yes*	251	184 (73.3)	246 *180 (73.2)*	83 *63 (35.0)*	0.590^b^	244 *178 (73.0)*	57 *41 (23.0)*	0.978^b^	243 *180 (74.1)*	34 *27 (15.0)*	0.579^b^
**Psychiatric disorder** *Yes*	351	53 (15.1)	297 *46 (15.5)*	101 *21 (45.7)*	0.100^b^	296 *46 (15.5)*	64 *23 (50.0)*	**<0.001** ^b^	282 *42 (14.9)*	36 *13 (31.0)*	**<0.001** ^b^
**Chronic Respiratory diseases** *Yes*	352	25 (7.1)	298 *22 (7.4)*	101 *10 (45.5)*	0.248^c^	297 *22 (7.4)*	64 *10 (45.5)*	**0.012** ^c^	283 *22 (7.8)*	36 *4 (18.2)*	0.500^c^
**Cardiovascular diseases** *Yes*	352	121 (34.4)	298 *100 (33.6)*	101 *31(31.0)*	0.535^b^	297 *100 (33.7)*	64 *30 (30.0)*	**0.018** ^b^	283 *100 (35.3)*	36 *15 (15.0)*	0.507^b^
**Overweight/obesity** *Yes*	336	190 (56.5)	289 *166 (57.4)*	96 *49 (29.5)*	0.154^b^	288 *165 (57.3)*	59 *35 (21.2)*	0.837^b^	276 *158 (57.2)*	34 *19 (12.0)*	>0.999^b^
**Hypertension** *Yes*	120	100 (83.3)	100 *82 (82.0)*	31 *24 (29.3)*	0.416^c^	100 *83 (83.0)*	30 *26 (31.3)*	0.772^c^	100 *81 (81.0)*	15 *12 (14.8)*	>0.999^c^
**Diabetes** *Yes*	352	43 (12.2)	298 *35 (11.7)*	101 *11 (31.4)*	0.890^b^	297 *36 (12.1)*	64 *14 (38.9)*	**0.013** ^b^	283 *33 (11.7)*	36 *5 (15.2)*	0.867^b^
Behavioural variables											
**Self-perceived knowledge about COVID19,** *Yes*	316	183 (57.9)	309 *181 (58.6)*	104 *63 (34.8)*	0.627^c^	308 *180 (58.4)*	64 *38 (21.1)*	0.888^c^	293 *172 (58.7)*	36 *23 (13.4)*	0.589^c^
**Self-perceived vulnerability to COVID19,** *Yes*	315	168 (53.3)	308 *165 (53.6)*	104 *66 (40.0)*	**0.018** ^b^	307 *164 (53.4)*	64 *40 (24.4)*	0.135^b^	292 *154 (52.7)*	36 *31 (20.1)*	**<0.001** ^b^
**Psychological support** *Yes*	314	108 (34.4)	307 *106 (34.5)*	102 *44 (41.5)*	**0.035** ^b^	306 *107 (35.0)*	64 *27 (25.2)*	0.224^b^	290 *100 (34.5)*	35 *18 (18.0)*	**0.039** ^b^
**Tobacco or e-cigarette smoking** *Yes*	317	56 (17.7)	309 *55 (17.8)*	103 *23 (41.8)*	0.157^c^	308 *55 (17.9)*	64 *11 (20.0)*	>0.999^c^	293 *52 (17.7)*	36 *8 (15.4)*	0.485^c^
Unhealthy alcohol use*** *Yes*	208	73 (35.1)	202 *73 (36.1)*	60 *25 (34.2)*	0.337^c^	201 *72 (35.8)*	41 *15 (20.8)*	>0.999^c^	192 *70 (36.5)*	18 *4 (5.7)*	0.211^c^
Mental health outcomes											
**Anxiety**	310	104 (33.5)									
**Depression**	309	65 (21.0)									
**Post-Traumatic Stress disorder**	294	36 (12.2)									

PLWHA, People living with HIV/AIDS.

*N: Not symptomatic, A: Mildly symptomatic, B: Moderately symptomatic, C: Severely symptomatic.

**Duration between COVID-19 diagnosis confirmation and enrolment.

***Audit-C score: ≥ 3 drinks for women and ≥ 4 for men.

a= t-test; b= Chi-square test; c= Fisher exact test. Bold refers to a p-value< 0.05.

Regarding clinical and biological characteristics, 54.8% were at stage A of the CDC classification for HIV infection, 64.7% had a CD4 count above 500 cells/mm^3^, and 8.2% had a detectable HIV RNA level (>50 copies/mL). The mean number (± SD) of self-reported symptoms was 7 (± 5) and 7.3% of the participants were newly re-infected with SARS-CoV-2. In addition, 13.8% of the participants were hospitalized. The majority of participants (56.5%) were overweight or obese: 34.4% had cardiovascular diseases; 12.2% had diabetes; and 7.1% had chronic respiratory diseases.

Regarding behavioral-related characteristics, 57.9% of the participants had a good knowledge of COVID-19, and 53.3% perceived themselves as vulnerable to COVID-19. Almost one-third of participants reported excessive alcohol use (35.1%), and 34.4% had received psychological support.

### Mental health outcomes

#### Anxiety

The prevalence of anxiety was 33.5% (95% CI [28.5%-39.0%]) (**Table 1**). The univariable analysis showed a lower prevalence of anxiety symptoms among participants who were professionally active (27.1% *vs*. 42.6%, p-value = 0.008). A higher prevalence of anxiety symptoms was reported among those who perceived themselves vulnerable to COVID-19 (40.0% *vs*. 26.6%, p-value = 0.018) and those who reported psychological support (41.5% *vs*. 28.9%, p-value = 0.035). In addition, those who reported a high number of symptoms were more likely to report anxiety symptoms (p-value < 0.001) (**Table 1**). In addition, those who reported anxiety symptoms also reported a high number of symptoms (10 (± 5) *vs* 7 (± 5); p-value < 0.001) (**Table 1**).

In the multivariable analysis (adjusted for sex, COVID-19 wave, and duration between COVID-19 confirmation and enrolment), older participants (aOR: 0.97; 95% CI [0.95-0.99]) and being professionally active (aOR: 0.43; 95%CI [0.25-0.75]), were significantly associated with lower odds of experiencing symptoms of anxiety. However, the number of COVID-19 symptoms was associated with higher odds of experiencing symptoms of anxiety (aOR: 1.17; 95%CI [1.11-1.24]) (**Table 2**).

**Table 2:** Factors associated with mental health outcomes among PLWHA co-infected with SARS-CoV-2 in France (logistic regression models, COVIDHIV cohort, n=371).

Participants characteristics	Anxiety				Depression				Post‐Traumatic Stress Disorder			
	OR [95% CI]	p-value	aOR [95% CI]^a^	p-value	OR [95% CI]	p-value	aOR [95% CI] ^b^	p-value	OR [95% CI]	p-value	aOR [95% CI] ^b^	p-value
Sociodemographic characteristics												
**Age (year)**	0.98 [0.96-0.99]	0.089	0.97 [0.95-0.99]	**0.010**								
**High school level or higher**	0.59 [0.29-1.18]	0.130			0.34 [0.17-0.72]	**0.004**			0.74 [0.30-2.11]	0.542		
**Professionally active**	0.50 [0.31-0.82]	**0.006**	0.43 [0.25-0.75]	**0.003**	0.35 [0.19-0.61]	**<0.001**	0.34 [0.18-0.65]	**0.001**	0.59 [0.29-1.21]	0.147		
**Born in metropolitan France**	0.86 [0.53-1.38]	0.524			0.70 [0.40-1.22]	0.209			0.81 [0.39-1.63]	0.556		
**Living in couple**	0.84 [0.52-1.36]	0.487			0.49 [0.28-0.86]	**0.014**	0.52 [0.2-0.98]	**0.043**	0.47 [0.22-0.96]	**0.042**	–	
HIV-related characteristics												
**Heterosexual HIV transmission**	0.95 [0.59-1.53]	0.835			0.90 [0.52-1.57]	0.722			1.20 [0.60-2.44]	0.609		
**Homosexual or bisexual HIV transmission**	0.90 [0.55-1.47]	0.681			0.78 [0.43-1.37]	0.388			0.60 [0.27-1.24]	0.183		
**Transfusion HIV transmission**	1.37 [0.18-8.42]	0.730			2.56 [0.33-15.76]	0.309			4.90 [0.63-30.60]	0.088		
**Drug injection HIV transmission**	2.15 [0.82-5.69]	0.116			3.30 [1.21-8.75]	**0.016**			1.68 [0.37-5.55]	0.437		
**CD4 count >=500cells/m^3^**	0.99 [0.60-1.65]	0.978			0.68 [0.39-1.21]	0.186			1.18 [0.57-2.59]	0.672	–	
**Detectable HIV viral load (>50 copies/mL)**	0.81 [0.30-1.95]	0.652			0.74 [0.21-2.05]	0.595					–	
CDC HIV clinical stage* *A* *B* *C*	1 1.85 [0.98-3.48] 1.04 [0.58-1.83]	0.054 0.900			1 2.03 [1.01-4.03] 0.86 [0.41-1.71]	**0.044** 0.677			1 1.59 [0.62-3.82] 1.22 [0.52-2.74]	0.311 0.638		
COVID-19-related characteristics and comorbidities												
**Hospitalization for COVID-19**	1.60 [0.73-3.43]	0.229			3.37 [1.51-7.38]	**0.002**	3.35 [1.34-8.33]	**0.009**	0.53 [0.08-1.91]	0.407		
**Received instructions at hospital discharge**	1.24 [0.68-2.31]	0.490			0.94 [0.49-1.85]	0.843	–		1.41 [0.61-3.68]	0.445	–	
**Number of COVID-19 symptoms**	1.15 [1.10-1.22]	**<0.001**	1.17 [1.11-1.24]	**<0.001**	1.14 [1.08-1.21]	**<0.001**	1.15 [1.08-1.22]	**<0.001**	1.26 [1.16-1.37]	**<0.001**	1.27 [1.16-1.41]	**<0.001**
**COVID-19 reinfection**	1.16 [0.34-3.63]	0.801			1.83 [0.47-5.97]	0.338			0.52 [0.03-2.80]	0.534	–	
**Psychiatric disorders**	1.80 [0.94-3.40]	0.072			5.10 [2.61-10.00]	**<0.001**			4.23 [1.90-9.19]	**<0.001**	4.04 [1.48-11.11]	**0.006**
**Respiratory diseases**	1.69 [0.69-4.07]	0.238			3.41 [1.37-8.32]	**0.007**			1.59 [0.44-4.59]	0.427	–	
**Cardiovascular diseases**	0.82 [0.49-1.37]	0.454			2.05 [1.16-3.62]	**0.013**			1.36 [0.66-2.76]	0.396	–	
**Diabetes**	0.88 [0.40-1.84]	0.743			2.69 [1.26-5.57]	**0.009**			1.26 [0.41-3.27]	0.656	–	
Behavioural variables												
**Self-perceived knowledge of COVID-19**	1.13 [0.70-1.84]	0.611			1.05 [0.60-1.85]	0.865			1.28 [0.63-2.71]	0.501	–	
**Self-perceived vulnerability to COVID19**	1.84 [1.14-3.01]	**0.013**			1.60 [0.91-2.84]	0.103			6.70 [2.75-20.12]	**<0.001**	4.53 [1.69-14.60]	**0.005**
**Psychological support**	1.75 [1.07-2.86]	**0.026**			1.48 [0.84-2.59]	0.175			2.23 [1.09-4.59]	**0.027**		
**Tobacco or e-cigarette smoking**	1.56 [0.85-2.83]	0.143			0.94 [0.44-1.90]	0.875			1.38 [0.56-3.12]	0.455		

a adjusted for sex, COVID-19 wave and duration between COVID-19 confirmation and enrolment.

b adjusted for age, sex, COVID-19 wave and duration between COVID-19 confirmation and enrolment.

*N: Not symptomatic, A: Mildly symptomatic, B: Moderately symptomatic, C: Severely symptomatic.

**Duration between COVID-19 diagnosis confirmation and enrolment.

***Audit-C score: ≥ 3 drinks for women and ≥ 4 for men. Bold refers to a p-value< 0.05.

#### Depression

In this study, almost one-fifth of the participants reported symptoms of depression (21.0%; 95%CI [16.8%-25.9%]) (**Table 1**). In the bivariate analysis, participants with high a school level or higher (17.6% *vs*. 38.5%, p-value = 0.006), those who were professionally active (13.8% *vs*. 31.6%, p-value < 0.001), or those living in a couple (15.5% *vs*. 27.1%, p-value = 0.020) were less likely to report symptoms of depression. While those who were hospitalized (43.3% *vs*. 18.5%, p-value = 0.004), who reported psychiatric disorders (50.0% *vs*. 16.4%, p-value < 0.001), chronic respiratory diseases (45.5% *vs*. 19.6%, p-value = 0.012), diabetes (38.9% *vs*. 19.2%, p-value = 0.013), or cardiovascular diseases (30.0% *vs*. 17.3%, p-value = 0.018), were more likely to report symptoms of depression. In addition, those who reported depression symptoms also reported a high number of COVID-19 symptoms (11 (± 5) *vs*. 7 (± 5); p-value < 0.001) (**Table 1**).

The multivariable analysis (adjusted for age, sex, COVID-19 wave, and duration between COVID-19 confirmation and enrolment) showed that being professionally active (aOR: 0.34; 95%CI [0.18-0.65]), and living in a couple (aOR: 0.52; 95%CI [0.2-0.98]) were significantly associated with lower odds of experiencing symptoms of depression. By contrast, being hospitalized for COVID-19 (aOR: 3.35; 95%CI [1.34-8.33]), and the number of self-reported symptoms were associated with higher odds of experiencing symptoms of depression (aOR: 1.15; 95%CI [1.08-1.22]) (**Table 2**).

#### PTSD

The prevalence of PTSD among the participants was 12.2% (95%CI [8.9%-16.5%]) (**Table 1**). The univariable analysis showed a lower prevalence of PTSD among participants living in a couple (8.5% *vs*. 16.5%, p-value = 0.047). a higher prevalence of PTSD was reported among female participants (17.8% *vs*. 9.3%, p-value = 0.040), those who perceived themselves vulnerable to COVID-19 (20.1% *vs*. 3.6%, p-value < 0.001) and those who reported psychological support (18.0% *vs*. 8.9%, p-value = 0.039). In addition, those who reported PTSD also reported a high number of symptoms (13 (± 5) *vs*. 8 (± 5); p-value < 0.001) (**Table 1**).

In the multivariable analysis (adjusted for age, sex, COVID-19 wave, and duration between COVID-19 confirmation and enrolment), perceiving oneself vulnerable to COVID-19 (aOR: 4.53; 95%CI [1.69-14.60]), the number of COVID-19 symptoms (aOR: 1.27; 95%CI [1.16-1.41]), and reporting psychiatric disorders (aOR: 4.04; 95%CI [1.48-11.11]) were associated with higher odds of reporting PTSD (**Table 2**).

## Discussion

This is one of the few studies conducted during the pandemic, providing real-world data on the dual burden of HIV and COVID-19, which may differ from post-pandemic research findings. In this study carried out over five waves of the COVID-19 pandemic in France, we have evaluated mental health outcomes among PLWHA coinfected by SARS-CoV-2. It provides important insight into the early psychosocial impact of COVID-19 among a group at potentially greater risk of negative health outcomes from this disease.

First, we found a high prevalence of mental health outcomes, including anxiety (33.5%), depression (21.0%), and PTSD (12.2%), among PLWHA coinfected by SARS-CoV-2 during the COVID-19 pandemic. Results from a large nationally representative survey among PLWHA in France (ANRS-VESPA2) in 2011 (31) revealed that depressive disorder prevalence was 28.1%, showing that the mental health of people living with HIV was a major concern even before the COVID-19 pandemic. In a recent study conducted in France among PLWHA, regardless of their COVID-19 status, Parisi et al. reported that 27.8% of the participants had moderate or severe depression symptoms at baseline (18). However, the prevalence of mental health in our study was found to be higher than that reported among the French general population at the start of the COVID-19 pandemic, particularly for anxiety symptoms (23.0%) (32) and depressive symptoms (13.5%) (33). These findings highlighted the negative impact of the COVID-19 pandemic, as a high proportion of PLWHA reported worse mental health outcomes. They may also support previous findings in the scientific literature that showed that PLWHA (whether or not coinfected with SARS-CoV-2) experienced an impairment of mental health during the COVID-19 pandemic (18, 34–37). In a recent study among PLWHA, 43% of the participants experienced symptoms of anxiety, and 14% met the threshold for anxiety disorders (34). A higher proportion of participants who reported feeling more anxious (77.6%) or more depressed (71.8%) than usual was also found in a study conducted in the United Kingdom (36). During the COVID-19 pandemic, access to mental health services, particularly for PLWHA, has been challenging in several countries. That could exacerbate the unmet mental health needs of this population, who may also face social violence due to their HIV status. As a result of the COVID-19 pandemic, PLWHA are disproportionately affected by fear of COVID-19 acquisition or complications and may experience greater psychosocial distress (38). These people may be in need of social support by engaging in social activities and other support that could help them cope with the negative effects of anxiety and stressors (20). It is important for healthcare providers to take a holistic approach to care while meeting the mental health needs of their patients. This may include counseling and support for mental health issues and ensuring access to HIV prevention and treatment services. Additionally, people living with HIV and COVID-19 can benefit from peer support groups that can provide a sense of community and connection during this difficult time.

Second, among the participants, poorer self-reported mental health outcomes were found to be significantly associated with the number of self-reported symptoms, self-perceived vulnerability to COVID-19, hospitalization for COVID-19, and psychiatric disorders.

Unsurprisingly, participants who reported a high number of self-reported symptoms also reported mental outcomes, including anxiety, depression, and PTSD, while those who were hospitalized because of COVID-19 were susceptible to being depressed. Patients with severe forms of COVID-19 may also have a higher number of symptoms that could lead to hospitalization, especially among individuals with underlying health conditions or compromised immune systems (39, 40). The coinfection of HIV/AIDS and COVID-19 could raise major challenges, including mental health concerns. Besides, hospitalization could be a stressful and overwhelming experience for anyone, particularly for PLWHA who are also dealing with COVID-19. There might be additional emotional and psychological challenges. Surviving a serious illness like COVID-19 and dealing with potential complications, including persisting COVID-19-related symptoms, may lead to mental health abnormalities (41, 42). In a cohort study, the post-acute medical and mental health burden among survivors hospitalized with COVID-19 was comparable to other acute infectious diseases (43). PLWHA might have concerns about their compromised immune systems and how COVID-19 could interact with their HIV/AIDS status. This could be especially relevant for PLWHA, who already might have experienced trauma related to their HIV diagnosis.

We observed that most participants perceived themselves as more vulnerable to contracting COVID-19 due to their HIV and COVID-19 status. Those participants were also more likely to report worse mental health, particularly PTSD. At the start of the COVID-19 pandemic, several studies had suggested that PLWHA were at greater risk of contracting COVID-19 and experiencing severe forms of the disease due to their HIV status (35–37), which could lead to a disruption of the immune system, making them more vulnerable. Although other studies subsequently failed to confirm these results, it was more than enough to scare PLWHA and make them aware of their possible vulnerability to COVID-19. That may strongly affect their mental health and contribute to added stress, particularly PTSD, as highlighted in our study and previous studies (44, 45). Moreover, in qualitative interviews, Hall et al. (46) found that the majority of PLWH reported having an increased perceived vulnerability to COVID-19 transmission due to HIV status, and that may have a negative impact on their lives, including HIV-related stigma and psychosocial issues. For this group, taking precautions, staying informed, and adhering to medical advice could significantly help to mitigate the risks associated with both HIV and COVID-19.

Third, in this study, better mental health outcomes were associated with older age, being professionally active, and living in a couple.

During the COVID-19 pandemic, older PLWHA may face challenges concerning mental health, as they may already be dealing with the effects of aging and managing a chronic illness, as well as the additional stressors of the COVID-19 pandemic that could have a significant impact on their mental well-being with an increase in anxiety about the risk of infection (47). However, in our study, the results show that the elderly seemed to manage their emotional health better, in particular their anxiety. That was consistent with the findings of other studies, which showed that the older PLWHA might be more vulnerable to severe illness if they contract COVID-19 due to their age and potential underlying health conditions and may experience psychological distress (47–50). It needs to reinforce the mental health or emotional support of older PLWHA during the COVID-19 pandemic by encouraging regular communication with healthcare providers to ensure access to medications and medical advice and engagement in stress-reducing activities such as gentle exercise and relaxation techniques.

Our findings highlighted that participants who were professionally active were less likely to report anxiety or depression symptoms. Maintaining good mental health among PLWHA who are also professionally active during the COVID-19 pandemic may be particularly challenging. Professional activities could help to manage some health concerns and cope with pandemic-related stressors that may require careful attention. Maintaining social connections and regular interactions with colleagues, friends, and family through virtual platforms, including online, could help reduce the stress of managing work and health needs (51–53). Encourage professionally active PLWHA coinfected with SRAS-CoV2 to provide flexible work arrangements such as remote work, adjusted schedules, or reduced workloads, which may be helpful to improve their mental health (54). They were also encouraged to seek individual counseling to address specific mental health concerns. Providing a supportive and understanding work environment could contribute significantly to the mental well-being of professionally active PLWHA during the COVID-19 pandemic.

As shown in this study, PLWHA coinfected with SRAS-CoV2 who were living in a couple relationship during the COVID-19 pandemic reported better mental health outcomes, particularly for depression symptoms. This result is in line with that of prior studies in the general population, which reported that those who were living alone frequently reported anxiety and/or depression symptoms (55, 56). Living in a couple relationship involves interaction dynamics that may impact individuals positively. Indeed, open communication between partners could help to share feelings, concerns, and any anxieties or depression they may have about COVID-19 and its impact on their health. That could also foster the development of a better environment in which they could understand and support each other’s emotions and worries.

The study focuses on a dual-infection population (PLWHA co-infected with SARS-CoV-2) who are often underrepresented in mental health research. By exploring various mental health outcomes (e.g., anxiety, depression, PTSD), the study provides a comprehensive analysis of the psychological impact of COVID-19 on PLWHA, a high-risk group.

However, there are some limitations in this study to consider when interpreting the results. All participants were recruited from HIV clinics across France that remained operational during the COVID-19 pandemic. However, our sample was not representative, and our results may not reflect the experience of all the PLWHA co-infected with SARS-CoV-2 in the country. The cross-sectional design of the present study is another limitation. Longitudinal studies are needed to further explore the impact of COVID-19 on mental health among PLWHA who got infected. In addition, data were collected among PLWHA at both the acute and chronic stages of COVID-19. Memory and desirability biases in self-reports also need to be taken into account, even if participants were informed that their responses would be analyzed in a non-judgmental way. The diagnostic of SARS-CoV-2 infection among PLWHA could introduce bias, as differences in the types of tests may affect results and interpretations. Despite these limitations, our findings provide a deeper insight into mental health issues among PLWHA during the COVID-19 pandemic.

## Conclusion

This large hospital-based study shows a high prevalence of anxiety, depression, and PTSD in PLWHA co-infected with SARS-CoV-2. Implementing strategies to prevent COVID-19 in this population could be crucial to improving mental health concerns. In addition, healthcare workers, including mental health professionals, should be aware of the potential mental health impacts of these disorders and provide comprehensive care for PLWHA coinfected with SARS-CoV-2, including addressing their mental health needs.

## Data Availability

The datasets presented in this article are not readily available because The data that support the findings of this study are available on request from MD. The data are not publicly available due to confidentiality restrictions. Requests to access the datasets should be directed to duracinsky.m@gmail.com.
